# Greyhounds under general veterinary care in the UK during 2016: demography and common disorders

**DOI:** 10.1186/s40575-019-0072-5

**Published:** 2019-06-04

**Authors:** Dan G. O’Neill, Nicola J. Rooney, Callum Brock, David B. Church, Dave C. Brodbelt, Camilla Pegram

**Affiliations:** 1 Pathobiology and Population Science, The Royal Veterinary College, Hawkshead Lane, North Mymms, Hatfield, Herts AL9 7TA UK; 2 Animal Welfare and Behaviour Group, Bristol Veterinary School, University of Bristol, Langford, BS40 5DU UK; 3 The Royal Veterinary College, Hawkshead Lane, North Mymms, Hatfield, Herts AL9 7TA UK; 4 Clinical Sciences and Services, The Royal Veterinary College, Hawkshead Lane, North Mymms, Hatfield, Herts AL9 7TA UK

**Keywords:** VetCompass, Electronic patient record, EPR, Breed, Dog, Epidemiology, Primary-care, Veterinary, Pedigree, Purebred, Racing

## Abstract

**Background:**

The greyhound is a sighthound known for its speed and agility. Greyhounds were selectively bred as functional racing animals but increasingly are kept as pets in the UK, often after their racing careers are over. The VetCompass™ Programme collates de-identified clinical data from primary-care veterinary practices in the UK for epidemiological research. Using VetCompass™ clinical data, this study aimed to characterise the demography, mortality and common disorders of the general population of pet greyhounds under veterinary care in the UK.

**Results:**

Greyhounds comprised 5419/ 905,544 (0.60%) dogs under veterinary care during 2016 from 626 clinics. Mean adult bodyweight was 29.7 kg (standard deviation [SD] 4.5 kg). Males (32.3 kg, SD 4.1 kg) were heavier than females (27.2 kg, SD 3.3 kg) (*P* < 0.001). Mean age was 7.6 years (SD 3.4). The most common colours were black (39.2%), black and white (20.8%), brindle (12.0%). Based on 474 deaths, median longevity was 11.4 years (range 0.2–16.5). Females (11.8 years) outlived males (11.2 years) (*P* = 0.002). The most common grouped causes of death were neoplasia (21.5%, 95% CI: 17.4–26.0), collapse (14.3%, 95% CI: 10.9–18.2) and musculoskeletal disorder (7.8%, 95% CI: 5.3–11.0). Based on a random subset of 2715/5419 (50.1%) greyhounds, 77.5% had > 1 disorder recorded during 2016. The most prevalent specific disorders were periodontal disease (39.0%, 95% CI: 37.2–40.9), overgrown nails (11.1%, 95% CI 10.0–12.4), wound (6.2%, 95% CI: 5.3–7.1), osteoarthritis (4.6%, 95% CI: 3.8–5.4) and claw injury (4.2%, 95% CI: 3.4–5.0).

**Conclusions:**

These findings highlight the greyhound as a relatively common pet dog breed in the UK, accounting for 0.6% of dogs under primary veterinary care. Dental disease, trauma and osteoarthritis were identified as common health issues within the breed. Knowledge of common disorders can help greyhound breeders and regulators to prioritise breeding, rearing and racing management to mitigate some of the most prevalent issues. Greyhound rehoming organizations can also better inform adopters about prophylactic care.

## Plain English Summary

Greyhounds were selectively bred as racing animals, but are increasingly kept as pets in the UK, often after their racing careers are over. Greyhounds have been reported in the veterinary literature with increased risk of 34 diseases but this does not necessarily mean that these diseases are either common or important for the breed. Using anonymised veterinary clinical information from the VetCompass™ Programme at the Royal Veterinary College, this study aimed to describe the demographic characteristics and the most common disorders of greyhounds under primary veterinary care in the UK.

Greyhounds comprised 5419 (0.60%) of the 905,544 study dogs. Males (32.3 kg) were heavier than females (27.2 kg). Overall, 77.5% greyhounds had at least one disorder recorded during 2016. The most common disorders recorded were dental disease (39.0%), overgrown nails (11.1%), wound (6.2%), osteoarthritis (4.6%) and claw injury (4.2%). Based on 474 deaths, the average lifespan was 11.4 years, with females (11.8 years) outliving males (11.2 years). The most common causes of death were cancer (21.5%), collapse (14.3%) and arthritis (7.8%).

The study documented the greyhound as a relatively common pet dog breed in the UK (0.6%) with a medium lifespan. Dental disease, injuries and osteoarthritis were highlighted as common health issues for the breed. These findings can provide useful evidence to greyhound breeders and regulators, ex-racing greyhound rehoming charities, to new owners and to veterinarians for prioritization of disease prevention and management in order to improve the health and welfare of greyhounds.

## Background

The greyhound is a sighthound known for its speed and agility [1]. Greyhound-type dogs, thought to be the prototype of sighthounds, were depicted on the walls of ancient Egyptian tombs. During the middle ages, greyhounds became popular throughout Europe, particularly with the nobility, for their hunting prowess [2]. The hunts (“coursings”) grew in popularity with the nobility during the eighteenth century in the British Isles, with attendance figures of up to 75,000 [3]. In 1926, the sport of racing greyhounds on an oval race track was first introduced to Britain from the USA [4, 5]. The sport has declined somewhat in recent years but remains in existence on the British Isles, the USA, Australia and China today [3, 6]. There are approximately 15,000 active racing greyhounds in the UK at present [7].

Most of the UK greyhound population were bred and raised in Ireland and were imported whilst racing. They are housed at individual “trainer’s” kennels, and the trainer is responsible for the care and husbandry of the dogs [8]. The majority of UK greyhound racing is regulated by the Greyhound Board of Great Britain (GBGB) who control 22 of the remaining 27 UK tracks. Greyhounds are not permitted to race at GBGB tracks until they are at least 15 months old, after which they race weekly, on average, before the majority retire from racing between the ages of three and five years [9, 10]. After retiring, most greyhounds are reportedly rehomed as pets [11], some directly by their trainers, but more often through a range of rehoming charities [10, 12]. The Greyhound Trust alone rehomes approximately 4000 retired greyhounds annually [13]. In contrast, there are relatively few greyhounds registered with the Kennel Club (KC) annually, with only 21 dogs newly registered during 2017 [14]. Therefore, the UK greyhound population is mainly divided into two distinct groups; younger racing greyhounds and the “pet” greyhound population which mainly consists of older, ex-racing animals [7]. Since designated veterinarians are legally required to attend all race meetings in England where 25 of the 27 UK tracks are located [15], it is likely that much routine and track-side emergency care is performed by the track veterinarian and hence may not feature in general veterinary practice data, whilst retired dogs are likely registered with routine primary care practices.

The pet greyhound is considered a medium-lived breed with a reported median longevity of 10.8 years compared with a median of 12.0 across all breeds [16]. However, despite their long history of selection for function (speed) rather than aesthetics [17], greyhounds have reported predispositions to 34 disorders [18] including osteosarcoma [19], ischaemic stroke related to systemic hypertension [20], chronic superficial keratitis (pannus) [21], cardiomegaly and left-sided systolic heart murmur [22, 23] and corns [24]. A US study of retired racing greyhounds reported that skeletal disease (32.5% prevalence) and skin disease (27.5% prevalence) were the most common disorder groups and that the most common cause of death was neoplasia (58% of all deaths with 42% of these neoplasia deaths being from osteosarcoma) [25].

Using veterinary clinical data from the VetCompass™ Programme [26], this study aimed to characterise the demography, longevity and common disorders of greyhounds under primary veterinary care in the UK. The study placed a special focus on disorders associated with age and sex. Although some evidence exists for sex and age predispositions to specific disorders in dogs overall [27, 28], there is limited information on sex and age associations within particular breeds [29–31]. There are currently strong opinions about reforming the racing greyhound industry in the UK. The GBGB has made a commitment that “Wherever possible, every dog leaving racing enjoys a long and happy retirement” [32]. The results from the current study could provide a reliable framework to assist such reforms [7, 33]. These results could additionally assist veterinary practitioners and owners with an evidence-base to help predict key health and welfare issues for pet greyhounds. This could in turn facilitate better education on prevention and early detection of disease to optimise the matching and care of retired racing dogs with adoptee homes.

## Materials and methods

The overall study population included all dogs under primary veterinary care at 626 clinics participating in the VetCompass Programme during 2016. Dogs under veterinary care were defined as having either a) at least one electronic patient record [EPR] (free-text clinical note, treatment or bodyweight) recorded during 2016 and/or b) at least one EPR recorded both before and after 2016. The VetCompass Programme collates de-identified EPR data from primary-care veterinary practices in the UK for epidemiological research [26]. Data fields available for VetCompass researchers include a unique animal identifier from each practice management system provider, species, breed, date of birth, colour, sex, neuter status and bodyweight, along with associated clinical information from free-form text clinical notes, VeNom diagnostic codes [34] and treatment with relevant dates. Demographic analyses included all 5419 greyhounds available in the study whereas disorder prevalence results were based on a random subset of 2715 (50.1%) of these greyhounds.

A cross-sectional study design derived from the cohort of 5419 greyhounds registered at participating practices was used to estimate the one-year period prevalence of the most commonly diagnosed disorders [35]. Sample size calculations estimated that disorder data needed to be extracted from at least 2651 greyhounds from the available study population of 5419 greyhounds to represent a disorder with 3.5% expected prevalence to a precision of 0.5% at a 95% confidence level [36]. Ethics approval was obtained from the RVC Ethics and Welfare Committee (reference number 2015/1369).

Dogs recorded as greyhound breed were categorised as greyhound and all remaining dogs were categorised as non-greyhound. *Adult Bodyweight* (Kg) described the mean from all bodyweight data for dogs aged over 18 months and was categorised into 6 groups (< 24.0, 24.0 to < 28.0, 28.0 to < 32.0, 32.0 to < 36.0, 36.0 to < 40.0, ≥ 40.0). N*euter* described the status recorded for the dog (entire or neutered) at a single time point 23rd May, 2018. *Age* described the age at the final date under veterinary care during 2016 (December 31st, 2016) and was categorised into 6 groups in line with some earlier breed studies [30, 37, 38] (< 3.0, 3.0 to < 6.0, 6.0 to < 9.0, 9.0 to < 12.0, 12.0 to < 15.0, ≥ 15.0).

The list of unique greyhound animal identification numbers was randomly ordered and the clinical records of a random subset were reviewed manually in detail to extract the most definitive diagnoses for all disorders recorded as present during 2016 regardless of whether these were the reasons for clinical presentation or were only detected later during the veterinary clinical examination [34, 39]. Elective (e.g. neutering) or prophylactic (e.g. vaccination) clinical events were not included. No distinction was made between pre-existing and incident disorder presentations. Disorders described within the clinical notes using presenting sign terms (e.g. ‘vomiting’ or ‘vomiting and diarrhoea’), but without a formal clinical diagnostic term being recorded, were included using the first sign listed (e.g. vomiting). Mortality data (recorded cause, date and method of death) were extracted on all deaths at any date during the available EPR data in the sample.

The extracted diagnosis terms were mapped to a dual hierarchy of precision for analysis: fine-level precision and grouped-level precision as previously described [39]. Briefly, fine-level precision terms described the original extracted terms at the maximal diagnostic precision recorded within the clinical notes (e.g. *inflammatory bowel disease* would remain as *inflammatory bowel disease*). Grouped-level precision terms mapped the original diagnosis terms to a general level of diagnostic precision (e.g. *inflammatory bowel disease* would map to *gastro-intestinal*).

Following data checking and cleaning in Excel (Microsoft Office Excel 2013, Microsoft Corp.), analyses were conducted using Stata Version 13 (Stata Corporation). The sex, neuter status, age, colour and adult bodyweight for greyhounds under veterinary care during 2016 were described. One-year (2016) period prevalence values were reported along with 95% confidence intervals (CI) that described the probability of diagnosis at least once during 2016. The CI estimates were derived from standard errors based on approximation to the binomial distribution [40]. Prevalence values were reported overall and separately for males and females. The median age (years) of cases on Dec 31st, 2016 was reported for fine-level and grouped-level disorders. Quantitative data were graphically assessed for normality and summarised with mean (standard deviation) or median (range) as appropriate [40]. The chi-square test was used to compare categorical variables and the Students t-test or Mann-Whitney U test to compare continuous variables as appropriate [40]. Statistical significance was set at the 5% level.

## Results

### Demography and mortality

**Fig. 1:**
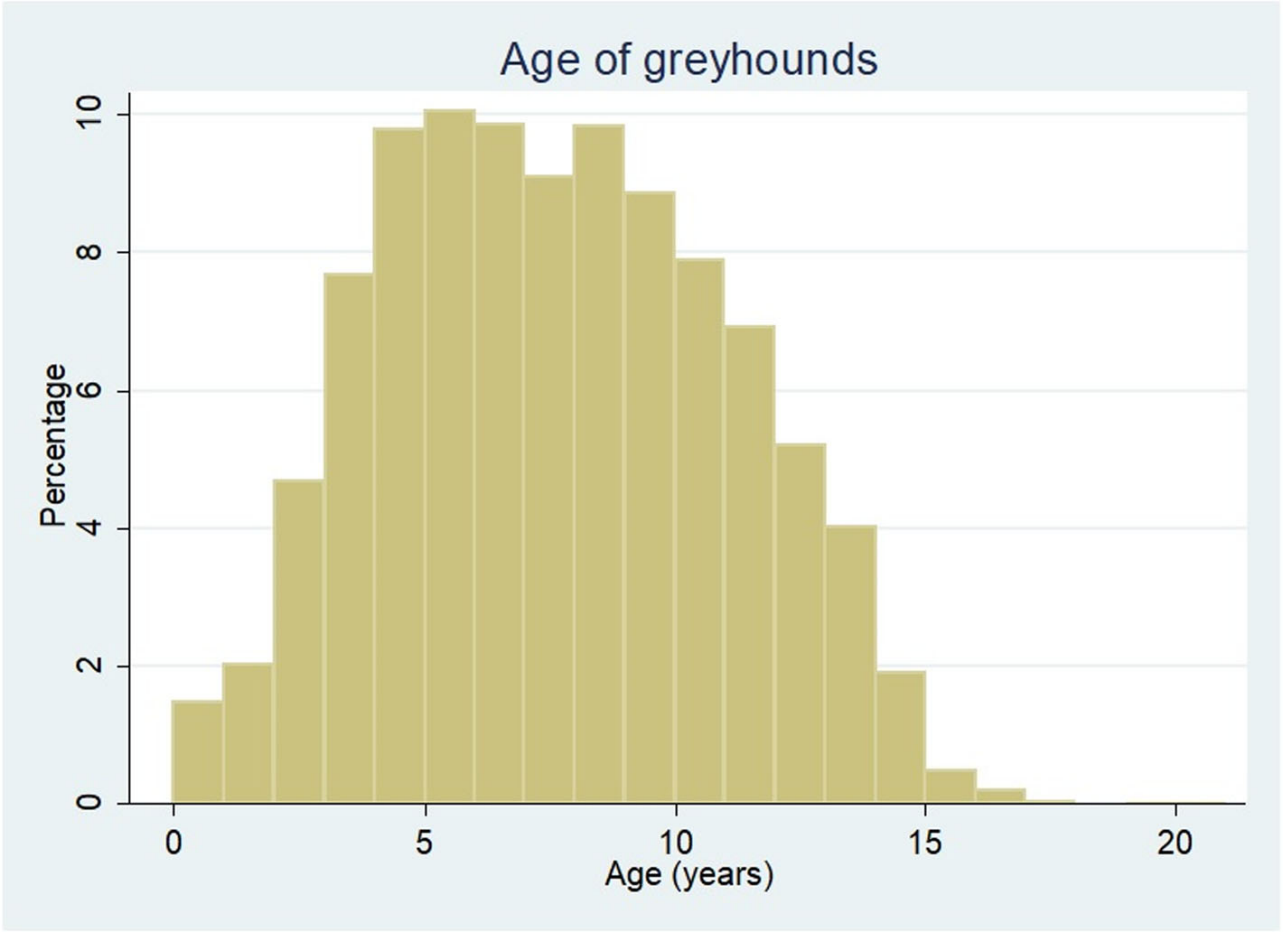
Ages (years) of greyhounds under veterinary care in the UK during 2016 at practices participating in the VetCompass Programme. (*n* = 5220)

The overall study population of 905,544 dogs from 626 clinics in the VetCompass database under veterinary care during 2016 included 5419 (0.60%) greyhounds. Of these 5419 greyhounds with information available, 2800 (51.9%) were female and 3671 (68.1%) were neutered. The probability of neutering did not differ between females (67.8%) and males (68.4%) (*P* = 0.642). The mean adult bodyweight overall was 29.7 kg (standard deviation [SD] 4.5 kg). The mean adult bodyweight of males (32.3 kg, SD 4.1 kg) was heavier than females (27.2 kg, SD 3.3 kg) (*P* < 0.001). The mean age of the greyhounds overall was 7.6 years (SD 3.4 (Fig. 1). Females (7.8 years, SD 3.5) were older than males (7.4 years, SD 3.3) (P < 0.001). There were 3570 (70.7%) dogs recorded with a single colour, 1473 (29.2%) recorded with two colours and eight (0.2%) with three colours. The most common colours overall were black (*n* = 1977, 39.2%), black and white (1049, 20.8%), brindle (606, 12.0%), blue (451, 8.9%) and fawn (386, 7.6%) (Table 1). Data completeness varied across the variables assessed: age 96.3%, sex 99.5%, neuter 99.5%, colour 93.2% and adult bodyweight 74.6%.

**Table 1:** Demography of 5419 greyhounds under primary veterinary care at practices participating in the VetCompass™ Programme in the UK from January 1st to December 31st, 2016

Variable	Category	Count^a^	Percent
Sex	Female	2800	51.9
	Male	2593	48.1
Female neuter	Entire	902	32.2
	Neutered	1898	67.8
Male neuter	Entire	820	31.6
	Neutered	1773	68.4
Female adult bodyweight (aged ≥18 months) (kg)	< 24.0	321	15.4
	24.0 to < 28.0	929	44.5
	28.0 to < 32.0	679	32.6
	32.0 to < 36.0	141	6.7
	36.0 to < 40.0	15	0.7
	≥ 40.0	1	0.1
Male adult bodyweight (aged ≥18 months) (kg)	< 24.0	45	2.3
	24.0 to < 28.0	192	9.8
	28.0 to < 32.0	632	32.4
	32.0 to < 36.0	748	38.3
	36.0 to < 40.0	276	14.1
	≥ 40.0	59	3.0
Age (years)	< 3.0	427	8.2
	3.0 to < 6.0	1436	27.5
	6.0 to < 9.0	1502	28.8
	9.0 to < 12.0	1236	23.7
	12.0 to < 15.0	580	11.1
	≥ 15.0	39	0.8

^a^Count covers dogs with available data

There were 474 deaths recorded during the study. The median longevity of greyhounds overall was 11.4 years (IQR 9.6–12.8, range 0.2–16.5). The median longevity of females (11.8 years, IQR 10.2–13.1, range 2.1–16.5, *n* = 236) was greater than males (11.2 years, IQR 9.1–12.5, range 0.2–16.0, *n* = 228) (*P* = 0.002). Median longevity did not differ between neutered (11.6 years, IQR 9.6–12.9, range 4.3–16.5, *n* = 338) and entire greyhounds (11.1 years, IQR 9.6–12.5, range 0.2–16.1, *n* = 126) (*P* = 0.289). The method of death was recorded in 463 (97.7%) of deaths. Of these, euthanasia accounted for 435 (94.0%) deaths while 28 (6.0%) were unassisted. Of the 372 (74.5%) deaths with a cause recorded, the most common causes of death described at a grouped-precision level were neoplasia (*n* = 80, prevalence 21.5%), collapse (53, 14.3%) and musculoskeletal disorder (29, 7.8%) (Table 2).

**Table 2:** Mortality in greyhounds with a recorded cause of death under primary-care veterinary at UK practices participating in the VetCompass™ Programme from January 1st to December 31st, 2016 (*n* = 372)

Grouped-level disorder	Count	Percent	95% CI
Neoplasia	80	21.5	17.4–26.0
Collapse	53	14.3	10.9–18.2
Musculoskeletal disorder	29	7.8	5.3–11.0
Mass-associated disorder	21	5.7	3.5–8.5
Spinal cord disorder	21	5.7	3.5–8.5
Thin/weight loss	17	4.6	2.7–7.2
Poor quality of life	15	4.0	2.3–6.6
Renal disease	14	3.8	2.1–6.2
Undesirable behaviour	12	3.2	1.7–5.6
Brain disorder	12	3.2	1.7–5.6
Enteropathy	11	3.0	1.5–5.2
Lethargy	11	3.0	1.5–5.2
Lower respiratory tract disorder	10	2.7	1.3–4.9
Traumatic injury	10	2.7	1.3–4.9
Other	56	15.1	11.6–19.1

### Disorder prevalence

The EPRs of a random sample of 2715/5419 (50.1%) greyhounds were manually examined to extract all recorded disorder data for 2016. There were 2103 (77.5%) greyhounds with at least one disorder recorded during 2016 while the remaining 22.5% had no disorder recorded and either presented for prophylactic management only or did not present at all during 2016. The median annual disorder count per greyhound during 2016 was one disorder (IQR 0–2, range 0–10). Median annual disorder count did not differ between females (1, IQR 1–2, range 0–8) and males (1, IQR 1–2, range 0–10) (*P* = 0.316).

The study included 4195 unique disorder events recorded during 2016 that encompassed 339 distinct fine-level disorder terms. The most prevalent fine-level precision disorders recorded were periodontal disease (*n* = 1060, prevalence 39.0%, 95% CI: 37.2–40.9), overgrown nails (302, 11.1%, 95% CI: 10.0–12.4), wound (167, 6.2%, 95% CI: 5.3–7.1), osteoarthritis (124, 4.6%, 95% CI 3.8–5.4) and claw injury (113, 4.2%, 95% CI 3.4–5.0). The median age of affected dogs varied from the youngest at 5.7 years for flea infestation to the oldest at 12.5 years for collapse (Table 3).

**Table 3:** Prevalence of the most common disorders at a *fine-level of diagnostic precision* recorded in greyhounds (*n* = 2715) attending UK primary-care veterinary practices participating in the VetCompass™ Programme from January 1st to December 31st, 2016

Fine-level disorder	Count	Overall prevalence %	95% CI^a^	Female prevalence %	Male prevalence %	Median age at end of study (years)
Periodontal disease	1060	39.0	37.2–40.9	39.4	38.8	8.3
Overgrown nails	302	11.1	10.0–12.4	11.8	10.5	8.3
Wound	167	6.2	5.3–7.1	5.8	6.6	6.8
Osteoarthritis	124	4.6	3.8–5.4	3.9	5.4	11.4
Claw injury	113	4.2	3.4–5.0	4.7	3.6	7.7
Diarrhoea	92	3.4	2.7–4.1	2.8	4.1	8.0
Lameness	84	3.1	2.5–3.8	3.0	3.2	7.7
Heart murmur	71	2.6	2.0–3.3	3.6	1.6	9.3
Corn	66	2.4	1.9–3.1	2.0	3.0	8.2
Dog-bite injury	55	2.0	1.5–2.6	2.3	1.8	7.4
Urinary incontinence	53	2.0	1.5–2.5	3.4	0.4	9.8
Aggression	47	1.7	1.3–2.3	1.0	2.6	7.1
Skin mass	45	1.7	1.2–2.2	1.1	2.3	8.5
Otitis externa	45	1.7	1.2–2.2	1.4	2.0	6.8
Collapse	44	1.6	1.2–2.2	1.8	1.5	12.5
Undesirable behaviour	41	1.5	1.1–2.0	1.9	1.1	6.5
Musculoskeletal injury	41	1.5	1.1–2.0	1.3	1.8	7.3
Foreign body	37	1.4	1.0–1.9	1.3	1.4	6.7
Stiffness	37	1.4	1.0–1.9	1.1	1.6	9.5
Noise phobia	36	1.3	0.9–1.8	1.6	1.1	7.6
Laceration	32	1.2	0.8–1.7	1.5	0.9	6.8
Flea infestation	32	1.2	0.8–1.7	1.0	1.4	5.7
Skin cyst	30	1.1	0.7–1.6	1.3	0.9	8.4
Urinary tract infection	29	1.1	0.7–1.5	0.9	1.3	9.2
Vomiting	29	1.1	0.7–1.5	1.1	1.1	9.2

^a^*CI* confidence interval

There were 53 distinct grouped-level precision disorder terms recorded. The most prevalent grouped-level precision disorders were dental (*n* = 1067, prevalence: 39.3%, 95% CI: 37.5–41.2), claw/nail (408, 15.0%, 95% CI: 13.7–16.4), musculoskeletal (368, 13.6%, 95% CI: 12.3–14.9) and traumatic injury (317, 11.7%, 95% CI: 10.5–12.9). The median age of affected dogs varied from the youngest at 5.6 years for parasitic conditions to the oldest at 10.8 years for underweight (Table 4).

**Table 4:** Prevalence of the most common disorders at a *grouped-level of diagnostic precision* recorded in greyhounds (n = 2715) attending UK primary-care veterinary practices participating in the VetCompass™ Programme from January 1st to December 31st, 2016

Grouped-level disorder	Count	Overall prevalence %	95% CI^a^	Female prevalence %	Male prevalence %	Median age at end of study (years)
Dental	1067	39.3	37.5–41.2	39.7	39.0	8.3
Claw/nail	408	15.0	13.7–16.4	16.0	14.1	8.1
Musculoskeletal	368	13.6	12.3–14.9	12.1	15.3	9.3
Traumatic injury	317	11.7	10.5–12.9	11.5	11.7	7.0
Skin/cutaneous	221	8.1	7.1–9.2	7.3	9.1	7.5
Enteropathy	202	7.4	6.5–8.5	6.3	8.7	7.9
Behavioural	169	6.2	5.3–7.2	6.3	6.3	7.6
Neoplastic	149	5.5	4.7–6.4	5.2	5.9	9.7
Ophthalmological	145	5.3	4.5–6.3	4.8	5.9	10.3
Urinary system	101	3.7	3.0–4.5	5.1	2.2	8.9
Mass-associated	88	3.2	2.6–4.0	2.9	3.7	9.3
Cardiac	88	3.2	2.6–4.0	4.4	2.0	9.5
Underweight	67	2.5	1.9–3.1	2.3	2.7	10.8
Parasitic	58	2.1	1.6–2.8	1.6	2.7	5.6

^a^*CI* confidence interval

## Discussion

This is the largest study to date that uses primary-care veterinary data to report on greyhound health, covering 5419 greyhounds under primary veterinary care in the UK. At 7.6 years, the mean age of greyhounds under primary care was quite old, likely because many were retired racers not presented to a primary-care practice during their earlier racing career. The median ages of Labrador Retrievers (4.9 years) [41], German Shepherd Dogs (4.7 years) [30] and Rottweilers (4.5 years) [37] from similarly designed studies were much younger. The most common causes of mortality were neoplasia, collapse and musculoskeletal disorder. The most prevalent disorders of greyhounds were periodontal disease, overgrown nails, wound, osteoarthritis and claw injury. These results reiterate the power of research based on large datasets of primary-care veterinary records to generate evidence and highlight key issues related to breed health in dogs [42]. These findings can contribute to more reliable frameworks for reforms in the UK greyhound industry, and also provide breeders, veterinary professionals and owners with an evidence-base to support their health and welfare activities for pet greyhounds [7, 33].

This median longevity of greyhounds in the current study was 11.4 years, which is higher than the median longevity of 10.8 years reported previously from a smaller group of primary-care greyhounds in England, but lower than reported longevity for the overall dog population across all breeds (12.0 years) [16]. Neutering is a time-dependent variable whereby the probability of being neutered increases (and cannot decrease) with age. However, neutering status information from most studies (and including the current study) should be interpreted with caution because these studies are often cross-sectional in design and include neutering as a time-independent binary variable that is generally taken as the neuter status at the time of death. Such analyses are prone to a reverse causality fallacy whereby greater longevity may promote increased probability of neutering but give the illusion that neutering promotes greater longevity [43]. Interestingly, the greyhounds under primary veterinary care in the current study may offer a solution to this time dependency issue because many are likely to have been rehomed as ex-racing dogs [10]. Given that most charities routinely neuter greyhounds before rehoming [13], many of the neutered dogs in the current study were likely to be neutered for their entire period within the current study. Consequently, this may result in the neuter variable in the current study behaving effectively as a time-independent variable and therefore offering more reliable analysis of neutering effects on longevity than studies in non-greyhound breeds. The current study identified no significant association between neuter status and longevity in greyhounds which contrasts with previous reports that neutered dogs across all breeds live longer than entire dogs [16, 44]. These results therefore do not support neutering as offering meaningful extension to longevity but does not rule out other substantial beneficial effects from neutering on quality of life or the risk of specific disorders. It is also possible that much of the health gains from neutering result from early-life neutering whereas neutering usually occurs later in post-racing life in greyhounds which could mitigate some of the putative benefits [28, 45].

The current study reports that female greyhounds outlive males (11.8 years versus 11.2 years respectively). This female longevity advantage is consistent with previous findings in some individual breeds including Rottweilers [37] and German Shepherd Dogs [30]. However, the female advantage may not be universal across all breeds and the true association between sex and longevity is likely to be complex, with many interacting factors as well as breed itself. A longevity analysis across all UK breeds identified equivalent longevity for entire and neutered males, with both groups outliving entire females by 0.4 years but being outlived by neutered females by 0.4 years [16]. An analysis of US referral data and UK primary-care data that specifically explored age differences between male and female dogs concluded that there were very limited sex effects on either longevity or causes of death in the companion dog [29]. A possible explanation for some of the apparent female longevity advantage in the current study may stem from differential ages at rehoming of post-racing greyhounds between the sexes. A genetic study evaluating the performance of racing greyhounds in Ireland identified a steeper performance decline after 40 months of age in females than in males [3]. This may result in later retirement of male greyhounds whose consequently longer racing careers might expose them to greater risk of injury and death both during this racing period and also afterwards because of potential cumulative persistent health damage [46]. However, these effects may also vary across female greyhounds depending upon whether their oestrus is pharmacologically supressed or not [9].

Neoplasia was the most common cause of death in greyhounds in the current study, accounting for 21.5% of deaths. This value is substantially lower than the value from a US online questionnaire of owners of retired racing greyhounds that reported neoplasia causing death in 58% of retired racing greyhounds, with osteosarcoma as the most common neoplasia type reported [25]. It is possible that the current study underestimated the total mortality from neoplasia by not including the 5.7% of deaths that were attributed to mass-associated disorders of which many may have truly been neoplastic in origin. Equally, however, it is also possible that the US online survey may have overestimated the proportional death rate by relying on owner-reporting which can be highly unreliable [47]. The 21.5% proportion of deaths from neoplasia reported here for greyhounds is slightly higher than the 16.5% neoplasia mortality reported across all breeds [16] and the 14.5% reported for German Shepherd Dogs [30] but substantially lower than the 33.0% reported for Rottweilers [37]. Collapse (14.3%) and musculoskeletal disorder (7.8%) were also common causes of death in greyhounds in the current study, in line with previous reports on greyhounds specifically, and also the wider dog population overall [16, 25].

Periodontal disease was by far the most prevalent fine-level disorder in the current study, with 39% of greyhounds affected during the single year of the study. This prevalence is more than four times higher than the 9.3% prevalence previously reported across all dog breeds [39] and also substantially higher than the 3.05% reported for Rottweilers [37] and 4.10% reported for German Shepherd Dogs [30] which are similarly large-sized breeds. Dental disease has been suggested to be more common in smaller sized dogs [48] which makes the exceptionally high prevalence in greyhounds all the more surprising. Greyhounds have been previously reported with a predisposition to some dental conditions [49] including progressive periodontitis [50] and supernumerary teeth [51]. As well as an intrinsic breed predisposition, rehomed racing greyhounds may also have predisposition to acquired dental disease because these dogs may receive limited veterinary and other dental care during their early rearing and throughout their racing careers resulting in poor dental hygiene [52]. It is also commonly asserted that early-life diet, and in particular soft food, may be contributory factors to poor dental health [9]. It is worth noting that the mean age of greyhounds in the current study was relatively old (7.6 years) and that, because periodontal disease progresses with age [53–55], the high prevalence of periodontal disease may be artifactually increased in this older population. However, despite this limitation, it can be reasonably concluded that periodontal disease is the most common clinical condition of greyhounds under primary veterinary care. This is important because dental disease can significantly compromise welfare, affecting the dog’s ability to eat and behave normally, as well as causing pain and discomfort [56] and being associated with other serious systemic conditions [57, 58]. Periodontal disease should be accepted as a significant issue within the breed that needs to be acknowledged and controlled. Preventive and remedial actions during the racing career of greyhounds could potentially reduce the incidence and severity of dental conditions both during this time and also positively impact on the dental health and overall welfare of these dogs after their racing careers are over [59]. A UK Government report on greyhound welfare highlighted poor dental health as a key financial constraint when rehoming racing greyhounds and stated that 14% of funds provided to the Greyhound Trust went on dental treatment [7]. In consequence, one of the recommendations from that report was that ‘*The industry should investigate whether poor dental health is prevalent in greyhounds and assess whether there are any measures that could be introduced to improve dental hygiene.’* [7]. To a large extent, the current study has answered the first question here and confirmed the importance of examining the opportunities raised by the second question. Recent research to explore opportunities to mitigate dental issues in racing dogs has highlighted the importance of dental care [9], but similar studies during the pre-racing and post-racing environment are still required.

Overgrown nails (11.1%), and claw injury (4.2%) were the second and fifth most commonly diagnosed conditions in greyhounds and showed higher prevalence than reported previously across all dog breeds: overgrown nails 7.1%, and claw injury 2.7% [39]. This suggests that nail-related disorders are both common and predisposed in greyhounds and therefore should be prioritised as important health issues in the breed. The reason for this predisposition is likely multifactorial. Ex-racing greyhounds may have sustained orthopaedic injuries during their racing careers [52], resulting in decreased later levels of activity and possible reluctance to exercise on hard surfaces. Therefore, this may contribute to the high prevalence of overgrown nails detected. If the nails are overgrown, they may subsequently be more prone to injury. Conversely, current racers and many ex-racing greyhounds tend towards sudden bursts of high activity which could predispose them to claw injury [60, 61]. Prevention of these health issues through vigilant monitoring by owners and veterinarians, regular nail trimming and detection of any underlying disease process is warranted.

Musculoskeletal disease was the third most common disorder group, with 13.6% of greyhounds affected during the study period. This was higher than the prevalence of 11.8% reported across all breeds [39]. At a more precise diagnostic level, osteoarthritis was diagnosed in 4.6% of greyhounds, higher than the estimated annual period prevalence of 2.5% reported across all breeds [62]. Risk factors associated with osteoarthritis diagnosis in the earlier study included being of higher bodyweight (i.e. a larger breed) and being older than eight years [62]. Greyhounds can be considered as a larger breed and many greyhounds in the current study were older than eight years, and thus the greyhounds in the current study had demographic risk factors that contributed to their risks for osteoarthritis. In addition, ex-racing greyhounds that had suffered fractures or other musculoskeletal injuries during their racing careers are more susceptible to post-traumatic osteoarthritis [63]. Therefore, osteoarthritis is another key health issue within the breed of which owners, particularly of ex-racing greyhounds with a history of orthopaedic injuries, and veterinarians should be aware. Osteoarthritis is a degenerative disease [62], and heightened awareness could promote the effective clinical management necessary to alleviate the associated pain and therefore protect animal welfare.

Traumatic injury was the fourth most common disorder group; 11.7% of greyhounds were affected by at least one event during the one-year period of the study which was higher than the 9.0% previously reported across all breeds [39]. At a finer level of diagnostic precision, wound was recorded in 6.2% and laceration was recorded in 1.2% of greyhounds in the current study. A predisposition to wounds and laceration in greyhounds may be attributed to their thinner skin which is more prone to damage compared to other breeds [64]. Additionally, ex-racing greyhounds may maintain a comparatively high chase drive and explosive activity after retirement with increased potential to increase the likelihood of self-inflicted injury [60, 61].

This study also suggests some sex-related differences in disorder prevalence for greyhounds that may be relevant for new or prospective owners to consider although the current study was relatively underpowered for reliable statistical comparisons of disorder occurrence between the sexes. Decision-making on whether to opt for a male or a female can be important for owners when rehoming a greyhound [13]. Female greyhounds appear to be more likely than males to be diagnosed with urinary incontinence (3.4% vs 0.4% respectively) and heart murmurs (3.6% vs 1.6% respectively). Urinary incontinence is widely accepted as being more common in female dogs than in males [65, 66], although the exact pathophysiological mechanisms are unclear [67, 68]. Heart murmurs in greyhounds, however, have not previously been identified as having a sex predisposition [22]. It is possible that this finding is partially explained by the increased longevity in females compared to males because the prevalence of heart murmurs rises steeply with aging in dogs [69] although there may be other explanatory factors that are as yet unknown. Skin masses appeared to be more prevalent amongst male than female greyhounds (2.3% vs 1.1% respectively). Although this is a novel finding, skin mass is not a very specific clinical entity and may refer to a number of cutaneous disorders, and therefore establishing the true relevance of this result would require a more detailed analysis with access to the precise diagnoses underlying each of these skin masses. Male greyhounds appeared to show higher prevalence of reported  aggression than female greyhounds in the current study (2.6% vs 1.0% respectively). A male predisposition to aggression is supported by a substantial body of evidence [37, 70, 71]. Aggression has been related to testosterone concentration and therefore the later neutering discussed above in this breed may a contributing factor. However, it should be noted that even in males, the recorded level of aggression constituted only one in forty dogs being reported aggressive which is much lower than reported previously in some other breeds such as the Rottweiler (7.46%) and German Shepherd Dog (4.76%) [30, 37]. Aggression is often fear-motivated and hence can constitute a welfare concern [72]. Multiple environmental and genetic factors contribute to the exhibition of aggressive behavior, and in greyhounds, this could result from suboptimal early socialisation or inappropriate transition from the racing to the retirement environment, where many new and potentially fear-provoking stimuli may be present. Most aggression is preventable [73] and treatable [74] which suggests that a focus on ways to mitigate these issues developing during rearing and at the time of rehoming may be beneficial. These suggestions of sex-based prevalence differences can be used for hypothesis generation for future confirmatory studies in order to contribute to improved greyhound health and welfare. Especially in relation to the possible aggression effects identified, future confirmatory analyses could assist rehoming centres to optimise their owner-selection procedures to ensure the best possible matching between the dogs and their new homes.

There are some limitations to the application of primary-care veterinary data for research that were relevant to the current study. In primary-care veterinary practice, a final specified diagnosis is not always reached, often due to the wishes or financial constraints of owners [75]. The current study attempted to manage this limitation by analysing grouped-level terms alongside fine-level terms [39]. As discussed above, the mean age of greyhounds in the current study was 7.6 years, which suggests that many animals entered the study population as adults and therefore the results may be skewed towards the disorders of older dogs. This also suggests that the younger (and possibly racing) subsets of the overall UK greyhound population may not be presenting to the veterinary groups in this study, as they are less likely to present at routine primary-care practices. Therefore, racing greyhounds and pet greyhounds can be considered as two distinct groups from a health perspective and the current results should be considered as applicable to the latter (mainly pet) population. The neuter data available for this study were based on the status at a single time point later than 2016. This meant that some dogs recorded as neutered in the study may have been entire during 2016 and therefore the results relating to neuter should be treated with some caution.

## Conclusion

This study of over five thousand greyhounds under general veterinary care highlighted the breed as relatively common in the UK. The greyhound was shown to be a medium-lived breed and neoplasia was identified as the most common cause of death. Periodontal disease was especially prevalent within the breed, and was therefore highlighted as a key health and welfare issue. Female greyhounds lived significantly longer than males. These results highlight some priorities that can be addressed by the greyhound industry during the breeding, rearing and active racing careers of these dogs [10], by greyhound rehoming charities and by new owners prior, during and after the rehoming process to optimise the welfare of the dogs.

## Abbreviations

CI: Confidence interval
EPR: Electronic patient record
GBGB: Greyhound Board of Great Britain
IQR: Interquartile range
KC: The Kennel Club
OR: Odds ratio
SD: Standard deviation
